# Objective measurements of the penile angulation are significantly different than self-estimated magnitude among patients with penile curvature

**DOI:** 10.1590/S1677-5538.IBJU.2017.0418

**Published:** 2018

**Authors:** Giovanni Liguori, Andrea Salonia, Giulio Garaffa, Giovanni Chiriacò, Nicola Pavan, Giorgio Cavallini, Carlo Trombetta

**Affiliations:** 1 Department of Urology, University of Trieste, Trieste, Italy; 2 Department of Urology, Università Vita-Salute San Raffaele, Milan, Italy; 3 Department of Urology, Università Vita-Salute San Raffaele, Milan, Italy; 4 Andrological Unit, Gynepro Medical Team; Bologna, Italy

**Keywords:** Penile Induration, Penis, Penile Erection

## Abstract

**Introduction:**

The study was aimed to assess the presence of actual differences between the objective and the perceived magnitude of a curvature between patients affected by Peyronie's disease (PD) and congenital penile curvature (CPC).

**Materials and Methods:**

Wee analysed a cohort of 88 consecutive patients seeking medi- cal help for either CPC or PD. All patients were invited to provide a self-made drawing of their penis in erection in order to obtain self-provided description of the deformity. An objective measurement of the deformity was also performed drawing two intersecting lines through the center of the distal and proximal straight section of the penile shaft.

**Results:**

Our findings showed significant differences between patient self-estimation and the objective measurements of the penile angulation performed by trained experts, with only 32% of patients correctly assessing their own curvature. Overall, patients tended to overestimate (56%) their degree of curvature, but the results are different in patients with PD than those with CPC. In the 60 men (68%) who did not accurately assess their curvature, PD patients generally overestimated their curvature versus CPC patients (67% vs 16%). On the contrary CPC patients underestimated their curvature compared to PD (42% vs. 4%).

**Conclusion:**

In order to improve patients' satisfaction rates, the surgeon needs to take into consideration the patient's perception of the deformity when planning the type of surgical correction.

## INTRODUCTION

Penile curvature can be classified as congenital (CPC) or acquired. Congenital penile curvature is secondary to an uneven development of the corpora cavernosa and usually becomes evident during adolescence, when the growth of the corporal tissue occurs at its maximum pace. Conversely, Peyronie's disease (PD) is an acquired benign connective tissue disorder involving the tunica albuginea of the corpora cavernosa, leading to the formation of fibrous inelastic plaques (1–4). As the fibrous inelastic plaques do not stretch as the rest of the tunica albuginea, during erections acquired curvature manifests with a variety of deformities including curvature, shortening, narrowing and hinge effect (1, 2).

The natural course of PD is not homogeneous and ranges from spontaneous resolution of all clinical symptoms to severe penile curvature, ED and the complete inability to engage in pene-trative sexual intercourse (1, 2, 4).

Surgery remains the mainstay of treatment for patients with CPC and stable PD and the aim of surgical correction is to guarantee a penis straight and rigid enough to allow the patient to resume penetrative sexual intercourse (1, 2). Unless the patient is troubled by refractory erectile dysfunction, the management of CPC is surgical straightening with the use of tunical plications (1). Tunical incision and grafting is almost never indicated in this group of patients as the curvature is harmonic and uniformly spread along the length of the shaft and hinge or hourglass deformities are not present. In patients with PD, the choice of the most suitable surgical intervention should be guided by an algorithm, which takes into consideration the quality of erections, the type and complexity of the curvature, the presence/absence of hourglass deformity and/or distal flaccidity and the degree of penile shortening (1, 2).

Although with an adequate choice of the surgical approach and in expert hands a satisfactory result can be achieved in most cases, patients' satisfaction rates following PD surgery tend to be significantly less encouraging (2, 5). To this regard, the surgical algorithm in PD surgery is based on an objective assessment by the surgeon of the degree of penile deformity (1, 2), but patient self-perception of the deformity can be different. It is in fact well documented that many patients complaining of PD tend to overestimate the degree of their curvature (6). Furthermore, the surgeon is only able to assess the deformity once PD is stable; on the contrary, the surgeon cannot actually appreciate the overall shortening that eventually has occurred. Likewise, usually there is not any morphometric pre-disease assessment which may help to objectively evaluate rates of penile malformations; therefore, the only “picture” of what morphometrically (i.e. shape, length, and curvature) the penis really was before the onset of PD is the one in the memory of the patient.

No previous studies have properly reported the relationship and differences in the perception of penile deformity between patients affected by PD and CPC.

We sought to i) assess the presence of actual differences between the objective (i.e. as assessed by the surgeon after induction of a penile artificial erection) and the perceived magnitude of a curvature among patients affected by PD and CPC; and, ii) correlate patient's self-perception of the degree of penile curvature with objective measures of the same angulation as obtained by trained specialists.

## MATERIALS AND METHODS

The analyses were based on a cohort of 88 consecutive Caucasian-European sexually active heterosexual men seeking medical help for either CPC or PD (either in the active or in the stable phase of the disease) between January 2013 and September 2014.

A comprehensive medical and sexual history, physical examination, and targeted laboratory and radiological [Dynamic Doppler Ultrasound (DDU) for patients with PD and penile plaque] investigations were performed in every patient (Table-1). Specific data collected included patient demographics, comorbidities, and regular medications. All patients were invited to complete the International Index of Erectile Function short--form (IIEF-5) (7) and the Beck's Inventory for depression (BDI) (8).

**Table 1:** Characteristics of participants.

		PD n. 69	CPC n. 19
**Erectile function**			
	EF (IIEF<21)	36	9
	EF (IIEF>21)	28	6
	No intercourse last 3 months	5	4
	PDE5i use	22	0
**Physical Examination(ICI)**			
	Curvature <30	9	5
	Curvature 30 - 60	29	7
	Curvature >60	31	7
	Palpable plaque	67	0
**PD treatment received**			
	Vitamine supplementation	26	N/A
	Intralesional injection (steroids or verapamil)	14	N/A
	ESWL	1	N/A
	No treatment	28	N/A
**Plaque US**			
	Isoechoic	18	N/A
	Hyperechoic	49	N/A
	Calcificated	2	N/A

Vascular parameters recorded with the DDU were within the normal reference range in all patients.

As part of the baseline office assessment, all patients were also invited to provide a self-made drawing of their penis in erection, both from an axial and a sagittal plane, in order to obtain self-provided tridimensional description of the deformity. Likewise, an objective measurement of curvature direction and angle was also performed drawing two intersecting lines through the center of the distal and proximal straight section of the penile shaft. The surgeon recorded both these measurements.

Axial and coronal photographs of the penis during a full pharmacologically induced erection were taken in the office setting by the surgeon during outpatient examination for every patient. In this context, a full erection was defined as the patient's impression of maximal achievable penile rigidity. Patients were initially injected with 10 mcg/mL of PGE1, which was eventually uptitrated to 20 mcg/mL until an adequate erection was eventually achieved.

**Figure 1:**
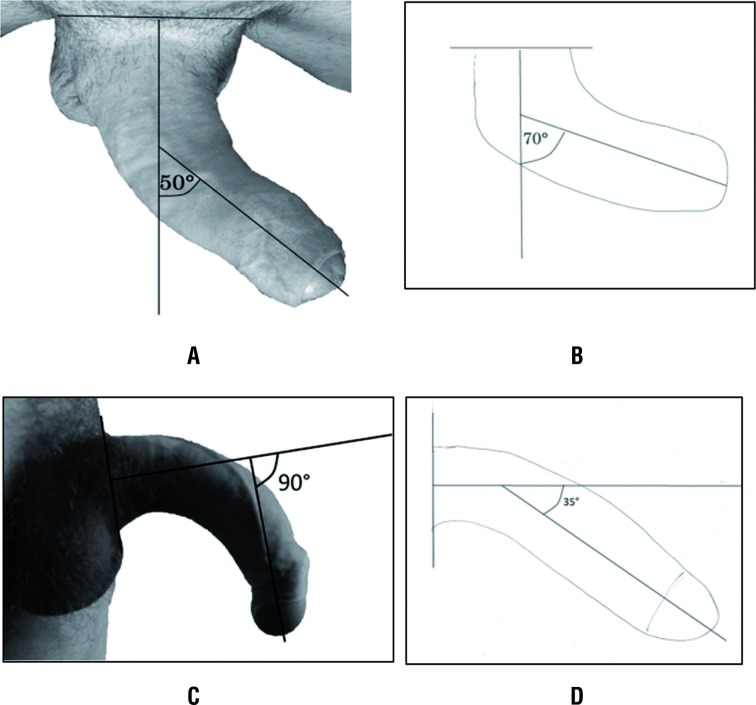
Example of overestimation of the penile curvature in a patient with PD. Objective measurement of the degree of penile curvature was performed on standardized photograph (A) and on the drawing; (B) of the same penis during erection using two intersecting lines. On the other hand, the drawings underestimate the curvature in a case of congenital penile deviation (C, D).

Using a ruler and starting at the base of the penis (proximal shaft), a straight line was drawn through the absolute center of the straight portion of the penile shaft proximal and distal to the point of maximum curvature. The degree of curvature was determined objectively measuring with a goniometer the angle between the two intersecting lines and recorded in the patient's notes (Figures 1A-D) (9).

Patients who failed to obtain a full erection in this experimental condition were excluded from this study. Similarly, patients who had complex multiplanar curvatures or significant associated deformities (hourglass, indentations) were excluded, as recording precisely the extent of the deformity was not completely reproducible.

For the specific purpose of the study, patients' perceived curvature and objective measurements were then finally compared; to this regard, patient's assessment was considered accurate if it fell within ± 5 degrees from the objective estimate of the surgeon.

Data collection was done following the principles outlined in the Declaration of Helsinki; all patients signed an informed consent agreeing to deliver their own anonymous information for future studies.

### Statistical analyses

Data are presented as median (standard deviation) unless otherwise indicated. Pearson co-efficient was performed to evaluate the correlation between the degree of deformity as self-perceived by the patient and the objective measurement. Chi-squared test was applied to evaluate rates between groups. A logistic regression analysis tested the correlation between the type/severity of the penile curvature and ED (as defined for IIEF-5 <21), as well as between the degree of curvature and potential curvature overestimation.

Statistical tests were performed using SPSS v.16 (IBM Corp., Armonk, NY, USA). All tests were two-sided, with a significance level set at 0.05.

## RESULTS

Table-2 details patient's characteristics as segregated according to the type of curvature. Overall, 69 (78.4%) patients had PD and 19 (21.6%) had CPC. The two groups did not differ in terms of penile curvature degree for physician assessment and IIEF score.

**Table 2:** Descriptive statistics of the whole cohort of patients [media (SD)].

	PD	CPC	p-value
No. of patients (%)	69 (78.4)	19 (21.6)	NA
Age (yrs)	54 (11.0)	26 (11.7)	<0.001
No. of patients with ventral curvature (%)	1 (1.4)	13 (68.4)	<0.001
No. of patients with lateral curvature (%)	22 (31.9)	5 (26.3)	0.03
No. of patients with dorsal curvature (%)	46 (66.7)	1 (5.3)	<0.001
Penile curvature (degree) Physician assessment	45 (17.8)	45 (24.9)	0.511
Penile curvature (degree) Patient assessment	60 (21.6)	35 (20)	0.002
Δ of measures (degree)	+15	-10	0.006
IIEF score	21 (6.6)	17 (8.3)	0.303
BDI-II score	6 (9.5)	4 (5.5)	0.07

**SD=**standard deviation; PD=Peyronie's disease; **CPC=**Congenital penile curvature; **IIEF=**International Index of Erectile Function; **BDI=**Beck's Inventory for depression

Table-3 lists clinical and disease's characteristics for PD patients.

**Table 3:** Clinical characteristics at presentation of patients with PD [No. of patients (%)].

Patients comorbidities		
Hypertension		19 (27)
Diabetes		12 (18)
Dupuytren		11 (16.4)
Stable sexual relationship		55 (80)
Length of the disease, months [median (SD)]		10 (22.3)
First clinical evaluation, months [median (SD)]		4 (10.8)
Pain	Flaccid	8 (12)
	Erection	26 (37)
	Coital	31 (45)
Inability to penetrate		32 (46)
Dyspareunia		18 (25.4)
Anxiety/stress		49 (71.6)

**PD =** Peyronie's disease; **SD =** standard deviation

**Figure 2:**
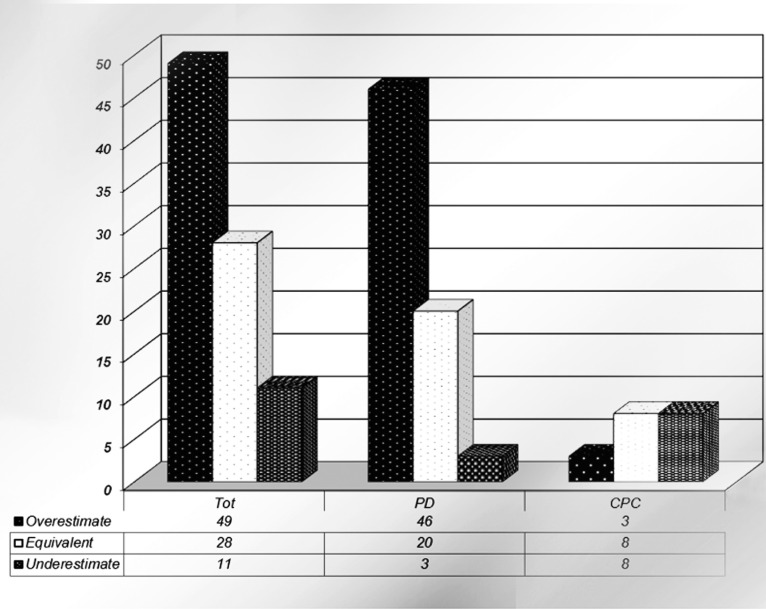
Rates of curvature estimation in the whole cohort, and as segregated for CPC and PD. Overall, most men with PD overestimated their curvature; conversely, men with CPC underestimated or provided a self-assessment of their curvature equivalent to that given by the physician.

The results of the comparison between patients' self-perception of the curvature and the objective assessment of the deformity are reported in Figure-2. Of all, 60 (80%) patients did not assess properly their own penile curvature; of them, 49 (81.6%) and 11 (18.4%) patients had PD and CPC, respectively. Within the PD group, 46 (93.9%) patients overestimated their curvature and 3 (6.1%) patients underestimated the curvature. Conversely, 8 (72.7%) patients with CPC underestimated the curvature and only 3 (27.3%) patients overestimated their curvature. Overall, 40 patients assessed properly their curvature, 20 (28%) in the PD group and 8 (42%) in the CPC group.

According to the statistical analysis, the mean patient perceived penile curvature at baseline was 45.97 (SD 19.4) degrees. However, the mean penile angulation obtained by objective measurement was 55.45 (SD 22.2) with a statistically significantly difference from the curvature perceived by the patient (p<0.05). The mean curvature at the patient subjective versus surgeon assessment was 59.06 degrees vs 45.43 degrees in the PD group and 42.37 degrees vs 47.89 in CPC group, respectively. The mean difference between the two measurements was: +13.48 (SD 16.2) degrees in PD group and -5.53 (SD 11.5) degrees in CPC group (p<0.01).

No differences were founded between PD and CPC by the degree of curvature according to the Kelami classification.

When stratified by the direction, differences between patients' subjective curvature and objective measures were significant for ventral and dorsal curvatures (p<0.05), but not for lateral.

Differences between patients perceived curvature and medical therapy were not significant.

Multivariate analysis revealed degrees of curvature (p=0.018) and IIEF score less than 21 (p=0.023 for IIEF <21) as independents predictors for patient's overestimation.

## DISCUSSION

Our findings clearly showed significant differences between patient self-estimation and the objective measurements of the penile angulation performed by trained experts, with only 32% of patients correctly assessing their own curvature. Overall, patients tended to overestimate (56%) their degree of curvature, but the results are different in patients with acquired curvature than those with congenital curvature. As a matter of fact, in the 60 men (68%) who did not accurately assess their curvature, PD patients generally overestimated than underestimated their curvature versus CPC patients (67% vs. 16%; p<0.005). On the contrary, CPC patients collectively underestimated their curvature compared to PD (42% vs. 4%; p<0.005).

The differences between patient estimates and the actual objective measures emerged to be significant for ventral and dorsal curvatures, but not for the lateral curvatures. At multivariate analysis, both the degree of curvature and the quality of erection, as psychometrically defined with the IIEF-5, emerged as independent predictors of patients' overestimation. Based on the results of our study, it could be postulated that the direction of curvature also matters: as a matter of fact in our series overestimation occurred in patients with dorsal curvatures. Unfortunately, the overwhelming predominance of the dorsal curvature in the population of PD patients limits this observation.

Strangely, in our series, the IIEF score of men with CPC was lower than PD. Although usually a better erectile function should be expected in men with CPC that are younger than PD, on the other hand, just because they are young and inexperienced and with a genital malformation, CPD patients may have a sexual problem.

Surgical correction of PD involves either shortening the longer aspect of the shaft with a plication-type procedure or lengthening the shorter aspect of the shaft with a relaxing incision followed by grafting. Conversely, penile implants are usually offered to patients with con-comitant ED (1, 2).

The aim of corrective surgery in PD is to guarantee adequate axial rigidity and to straighten the penis enough to allow the patient to resume penetrative sexual intercourse (1, 2). As stated, notwithstanding a successful surgical correction can be achieved with an adequate choice of the more tailored surgical approach; patients' satisfaction following PD surgery is not very high (1, 2, 5, 10, 11). For instance, Akin-Olugbade et al. analyzed patients satisfaction rates in a series of 114 patients submitted to penile implants; they reported that men diagnosed with PD had lower satisfaction rates when compared with the general penile implant population, possibly because of the more significant penile shortening experienced by this specific subset of patients (5). These findings have been confirmed by Kueronya et al., who have shown how subjective loss of penile length preoperatively is reported by most patients and that further penile length loss due to surgical correction could lead to a significant bothersome condition, irrespectively of the mag-nitude of the loss itself (1, 12).

We hypothesized that the reason for the low patients' satisfaction rate could be the different self-perception of the deformity. As a matter of fact, the main limitation of the surgical algorithm for PD is that the choice of the most suitable procedure is based on an objective assessment by the surgeon of the degree of penile deformity, but patient self-perception of the deformity can be different (6). Furthermore, the surgeon is actually able only to assess the deformity produced by PD once the active phase of the disease is over, but cannot appreciate the overall shortening and contracture that has occurred since the onset and throughout the natural evolution of the disease.

To this regard, findings are in contrast with the result of a previous series of Matsushita et al. where only 16% of patients did overestimate the deformity (13). Those findings of Matsushita et al. may be explained by the fact that patients had assessed subjectively their curvature during a spontaneous erection rather than with a pharmacologically induced one. Indeed, a full pharmacologically induced erection would have produced a more significant stretch of the tunica albuginea, when compared with a normal erection, and this would have rendered the deformity more obvious.

Therefore, strength of our findings is the fact that in the present series the deformity has been assessed both by the patient and the surgeon after an intracavernosal injection, in order to minimize potential measurement bias during a spontaneous erection, especially if the rigidity was suboptimal.

Similarly, we decided neither to use self-photography of the erect penis to assess the deformity, nor a vacuum constriction device to obtain an erection because they can significantly underestimate the degree of the curvature, as the quality of the erection produced may be suboptimal (14, 15).

From a clinical standpoint, since men with PD may have poor body image leading to mood disturbances, low self-esteem, and emotional distress, any effort aiming to better understanding patients' expectations should be welcomed (16, 17). This could eventually lead to a different approach to treatment and to the creation of a new surgical algorithm, which will take more into consideration patient perception of the deformity and of the loss of sexual function and may lead to better patients' satisfaction postoperatively.

Moreover, our results seem to show that patients with PD significantly overestimate their curvature than patients with congenital curvature, which, on the contrary, generally underestimate it. Although the reason of this difference is not known and is not the object of this study, we speculated that, if the deformity appears after the patient's self-image has already been well defined, the perception of self-reported magnitude is worse. Otherwise, a patient with a congenital curvature made eye to his penis during the growing and his curvature was self-recognized from their early teens with the development of self-sexual awareness, showing minor psychological implication. Moreover, as opposed to PD patients, it is observed that CPC patients have mainly a ventral curvature and one wonders whether this direction of curvature is exactly the variable of concern. Unfortunately in this group of patients, only 1% presents a dorsal curvature and for this reason a correlation is not viable.

This study is not devoid of limitations that the reader should be aware of and may limit the conclusions drawn from these data. First, this study lacks an appropriate comparison group. It's likely that the limited numbers of congenital penile curvature patients with dorsal curvatures and, similarly, the small number of patient with ventral curvature in the Peyronie's disease patient group, limits any kind of conclusion about whether patients in this population might otherwise overestimate or underestimate their curvatures in this scenario. A second obvious limitation pertaining the fact that our series includes only patients presenting for clinical evaluations, rather than a wider group of patients with PD.

It is likely that patients presenting for clinical management are more distressed about their conditions and are prone to overstate the problem. Lastly, this study did not include the assessment of partners, did not collect data on “risk factors” and was limited in the number of demographic variables assessed. In light of these criticisms and the results of this study, a longitudinal study with a standard baseline and regular assessment is warranted to confirm or contrast the data presented in this manuscript. In addition, future studies that assess the impact on the partner and include an appropriate comparison group would be useful in determining the impact of PD on the patient and the couple.

## CONCLUSIONS

Adequate preoperative counselling is extremely helpful to give patients more realistic expectations in terms of surgical outcomes. In this context, because it is well documented that PD may lead to depression, low self-esteem and relationship difficulties, we assessed the potential psychological impact of the disease and depression-related symptoms with self-reported BDI-II (1, 2, 10). To this specific purpose we invited all patients to fill the BID-II in; of clinical relevance, no correlation was observed between the self-assessment of the morphometric alte-ration of the penile shaft and a potential mood deflection. Conversely, our findings confirmed that, since a complete restoration of what the penis used to look like before the onset of PD is not possible, in order to further improve patients' satisfaction rates, the surgeon needs to take into consideration a patient's perception of the deformity and of the shortening of the penis which is most likely close to the reality during the decision making process and certainly when planning the type of surgical correction (10).

## ABBREVIATIONS

CPC: = Congenital penile curvature
PD: = Peyronie's disease
ED: = Erectile dysfunction
DDU: = Dynamic Doppler Ultrasound
IIEF-5: = International Index of Erectile Function short-form
BDI: = Beck's Inventory for depression
